# CT295 Is *Chlamydia trachomatis’* Phosphoglucomutase and a Type 3 Secretion Substrate

**DOI:** 10.3389/fcimb.2022.866729

**Published:** 2022-06-20

**Authors:** Sébastien Triboulet, Maimouna D. N’Gadjaga, Béatrice Niragire, Stephan Köstlbacher, Matthias Horn, Vishukumar Aimanianda, Agathe Subtil

**Affiliations:** ^1^ Institut Pasteur, Université Paris Cité, CNRS UMR3691, Unité de Biologie Cellulaire de l’Infection Microbienne, Paris, France; ^2^ Sorbonne Université, Collège Doctoral, Paris, France; ^3^ Centre for Microbiology and Centre for Microbiology and Environmental Systems Science, University of Vienna, Vienna, Austria; ^4^ Institut Pasteur, Université Paris Cité, CNRS UMR2000, Unité de Mycologie Moléculaire, Paris, France

**Keywords:** *Chlamydia*, glycogen, metabolism, type 3 secreted effectors, phosphoglucomutase (PGM), secretion signal

## Abstract

The obligate intracellular bacteria *Chlamydia trachomatis* store glycogen in the lumen of the vacuoles in which they grow. Glycogen catabolism generates glucose-1-phosphate (Glc1P), while the bacteria can take up only glucose-6-phosphate (Glc6P). We tested whether the conversion of Glc1P into Glc6P could be catalyzed by a phosphoglucomutase (PGM) of host or bacterial origin. We found no evidence for the presence of the host PGM in the vacuole. Two *C. trachomatis* proteins, CT295 and CT815, are potential PGMs. By reconstituting the reaction using purified proteins, and by complementing PGM deficient fibroblasts, we demonstrated that only CT295 displayed robust PGM activity. Intriguingly, we showed that glycogen accumulation in the lumen of the vacuole of a subset of *Chlamydia* species (*C. trachomatis*, *C. muridarum*, *C. suis*) correlated with the presence, in CT295 orthologs, of a secretion signal recognized by the type three secretion (T3S) machinery of *Shigella*. *C. caviae* and *C. pneumoniae* do not accumulate glycogen, and their CT295 orthologs lack T3S signals. In conclusion, we established that the conversion of Glc1P into Glc6P was accomplished by a bacterial PGM, through the acquisition of a T3S signal in a “housekeeping” protein. Acquisition of this signal likely contributed to shaping glycogen metabolism within *Chlamydiaceae*.

## Introduction

Many bacteria and parasites develop inside vacuolar compartments within eukaryotic cells. Such enclosed replication niches provide a shelter against extracellular and cytosolic host defense. They can also be further exploited to sequester cytoplasmic components and make them accessible only to the intruder. One striking example of this behavior is the vacuole in which the human adapted pathogen *Chlamydia trachomatis* grows, also called the inclusion (Elwell et al., 2016). Glucose is the main carbon source for these obligate intracellular bacteria (Harper et al., 2000; Iliffe-Lee and McClarty, 2000; Nicholson et al., 2004; Mehlitz et al., 2017). Over the course of their developmental cycle, they hijack a considerable amount of glucose from their host, and accumulate it as a polysaccharide, i.e. glycogen, in the lumen of the inclusion (Gordon and Quan, 1965; Chiappino et al., 1995). The *C. trachomatis* genome encodes for all the enzymes necessary for a functional glycogenesis and glycogenolysis, a surprising observation given that this pathway is absent in most intracellular bacteria (Henrissat et al., 2002). Glycogen starts accumulating in the lumen of the inclusion about 20 hours post infection (hpi), a time of intensive bacterial replication (Gehre et al., 2016). Shortly later, while luminal glycogen continues to accumulate, a fraction of the dividing bacteria, called reticulate bodies (RB), initiate conversion into the non-dividing, infectious, form of the bacterium, the elementary body (EB). Electron microscopy studies showed that, in contrast to RBs, EBs contain glycogen, but in modest quantities compared to the luminal glycogen. While part of the luminal glycogen is imported in bulk from the host cytoplasm, the majority is synthesized *de novo* through the action of bacterial enzymes (Gehre et al., 2016). Bacterial glycogen synthase GlgA, and branching enzyme GlgB, have acquired type 3 secretion (T3S) signals, recognized by the T3S of *Shigella flexneri*, that allow their secretion in the lumen of the inclusion, where they ensure glycogen synthesis out of UDP-glucose imported from the host cytosol (Gehre et al., 2016).

Bacterial enzymes responsible for glycogen catabolism into Glc1P, i.e. the glycogen phosphorylase GlgP, the debranching enzyme GlgX, and the amylomaltase MalQ, have also acquired secretion signals recognized by the T3S machinery of *S. flexneri* (Gehre et al., 2016). In *C. trachomatis* infection, they are expressed as early as 3-8 hpi, indicating that even if glycogen is only detected 20 hpi, there is a continuous flux of Glc1P generation in the inclusion lumen earlier on (Belland et al., 2003; Gehre et al., 2016). However, Glc1P cannot sustain bacterial growth directly because the glucose transporter UhpC can only import Glc6P, not free glucose nor Glc1P (Schwoppe et al., 2002; Gehre et al., 2016). Thus, while the rate of expression and distribution of *C. trachomatis* glycogen enzymes and the strategy of glycogen storage in the inclusion lumen by RBs point to a sophisticated pathway for hijacking host glucose, this metabolite would not be able to fuel bacterial metabolism due to a lack of an import mechanism.

To solve this conundrum, we had hypothesized that an enzyme with PGM activity was present in the inclusion lumen (Gehre et al., 2016). In the present work we tested this hypothesis in depth by investigating two non-mutually exclusive possibilities as to its origin: the translocation of a host enzyme into the inclusion, and the secretion of a bacterial enzyme. We provide evidence for the second hypothesis and identify CT295 as *C. trachomatis* PGM. In addition, we show that this feature is characteristic of the glycogen accumulating *Chlamydiaceae*, as opposed to the species that do not store glycogen in their inclusions.

## Results

### Absence of Evidence for the Import of Host Phosphoglucomutase in the Inclusion Lumen

**Figure 1:**
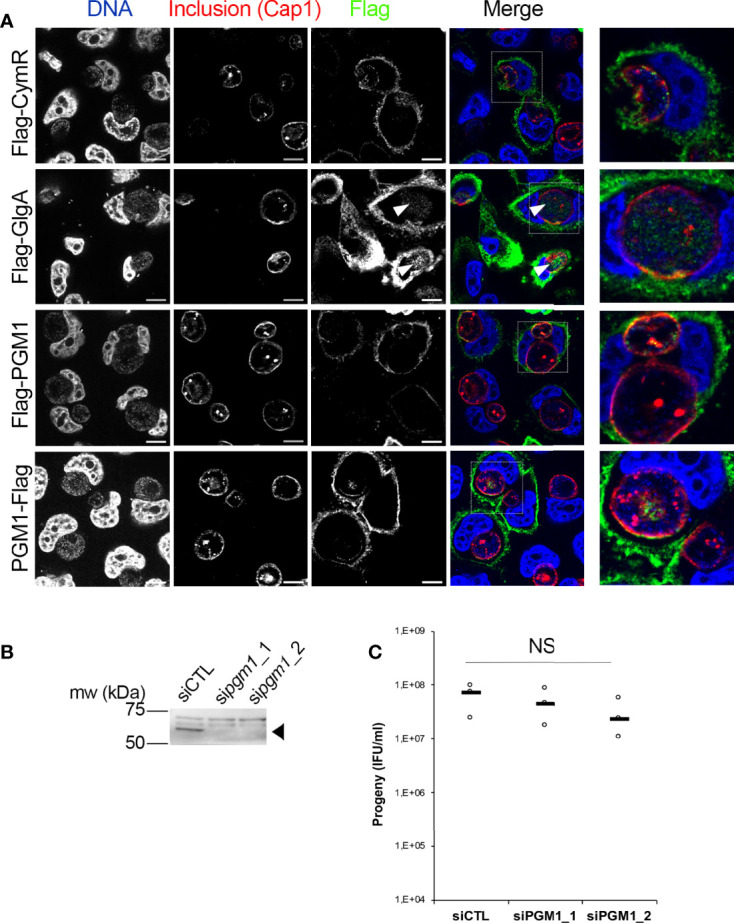
Host PGM1 does not accumulate in the inclusion lumen and is dispensable for *C. trachomatis* development. **(A)** – HeLa cells were transfected with the indicated Flag-tagged constructs, infected with *C. trachomatis* L2 and fixed 24 h later. Cells were permeabilized and stained for the inclusion membrane protein Cap1 (red) and Flag (green). DNA was stained with Hoechst (blue). The merge image is shown. An enlargement of the boxed area is displayed on the right. Only Flag-GlgA was consistently detected in the inclusion lumen (arrowheads). Bar is 10 μm. **(B)** – Two different siRNA were used to silence *pgm1* expression and the effect on PGM1 level was measured by western blot. The arrowhead points to PGM1 (mw=61.5 kDa), higher bands are nonspecific. **(C)** – The effect of *pgm1* silencing on bacterial progeny was measured 48 hpi. Dot plot distribution of the progeny for three independent experiments is shown. Gray bar, median. Statistical significance was calculated using two-way analysis of variance (ANOVA) with Dunnett’s multiple comparison test and was not significant (NS).

PGM activity in human cells is carried out by the enzyme PGM1. PGM1 deficiency leads to defects in glycogen storage, and to several disorders resulting from insufficient protein glycosylation (Stojkovic et al., 2009; Tegtmeyer et al., 2014). To test for the presence of this enzyme in *C. trachomatis* inclusions we expressed PGM1 with either an amino-terminal (Flag-PGM1) or a carboxy-terminal (PGM1-Flag) Flag tag in HeLa cells, which were further infected with *C. trachomatis* L2. The cells were fixed 24 h later and stained with a mouse antibody against the Flag tag and a rabbit antibody against the inclusion protein Cap1. An irrelevant Flag-tagged protein (Flag-CymR) was included as a negative control, and Flag-tagged chlamydial glycogen synthase (Flag-GlgA), as a positive control (Gehre et al., 2016). Flag-CymR, Flag-PGM1 and PGM1-Flag had a similar distribution (**Figure 1A**). Only very few dots could be observed in the inclusion lumen. In places where a more substantial overlap with the inclusion was observed, it was in areas positive for Cap1 staining, suggesting a cytoplasmic distribution adjacent to the inclusion membrane (see for instance the PGM1-Flag staining in **Figure 1A**).In contrast, as previously reported, we could detect Flag-GlgA in the inclusion lumen, presumably because it is co-transported with host glycogen into the inclusion lumen (Gehre et al., 2016). One caveat of this experiment is that Flag-GlgA was expressed at higher level compared to the other three constructs, which facilitates its detection in the inclusion lumen. Altogether, these observations indicate that PGM1 is not translocated in the inclusion lumen. We next tested whether silencing PGM1 expression had any effect on bacterial development. Cells were infected 24 h after siRNA transfection with two different siRNA (si*pgm1*_1 and si*pgm1*_2), and the resulting progeny was measured. The efficacy of each of the siRNA against PGM1 was validated by western blot (**Figure 1B**). None of the two siRNAs tested had a significant effect on the progeny collected 48 hpi (**Figure 1C**). We concluded from this series of experiments that host PGM1 does not reach the inclusion lumen, at least not in detectable amount, and is not essential for the generation of infectious bacteria. Therefore, it is unlikely that the conversion of Glc1P into Glc6P in the inclusion lumen relies on the host PGM activity by PGM1.

### CT295 Is the Main PGM in *C. trachomatis*, While CT815 Displays Low Activity Towards Glc1P

**Figure 2:**
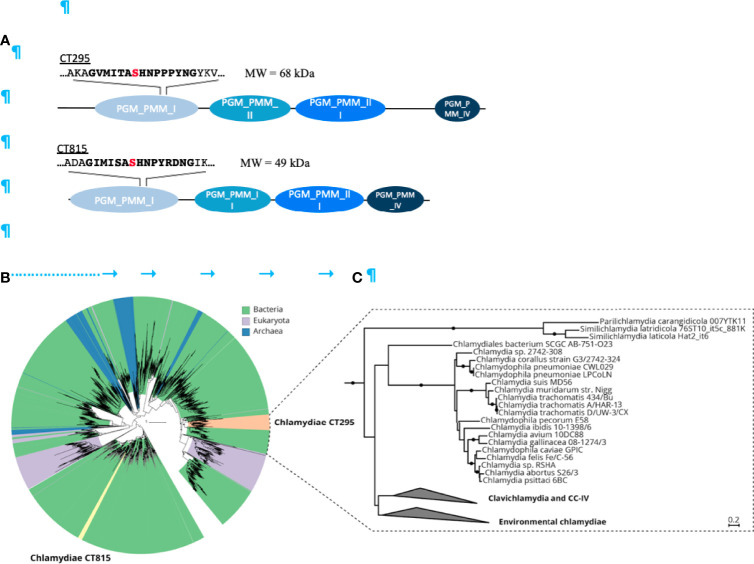
The chlamydial candidate PGMs CT295 and CT815 are related with other bacterial PGMs, stem from an ancient gene duplication event and are both monophyletic. **(A)** – Schematic representation of the two putative PGMs from *C. trachomatis*. PGMs are composed of four phosphoglucomutase domains depicted in blue. The PGM conserved motif sequence is in bold with the serine involved in the phophoryl-transfer in red. **(B)** – Phylogenetic analysis of chlamydial CT295 and CT815 homologs in eggNOG COG1109 (annotated as “phosphomannomutase”; 456 aligned positions) clustered at 80% amino acid identity over 90% query coverage (n = 2,829). Clades are colored by their affiliation to bacteria, eukaryota, and archaea in green, violet, and blue, respectively. Orange indicates the chlamydial CT295 clade (bacterial eggNOG ENOG4107QSU), and yellow indicates the chlamydial CT815 clade (bacterial eggNOG ENOG4107QJF). Maximum likelihood phylogenetic trees with best fit model LG+C60+G+F with 1,000 ultrafast bootstraps are shown. Bootstrap support for monophyly of chlamydial clades in the tree is ≥ 95%, and the SH-like approximate likelihood ratio is ≥ 80%. **(C)** – Phylogenetic analysis of chlamydial CT295 homologs (bacterial eggNOG ENOG4107QSU; n = 104) with its sister clade from COG1109 (575 aligned positions) used as outgroup (n = 93). Maximum likelihood phylogenetic tree based on LG+C60+G+F best tree using the posterior mean site frequency model and 100 non-parametric bootstraps. Bootstraps ≥ 70% are indicated by filled circles at splits in the tree. Scale bar indicates 0.2 substitution per position.

CT295 and CT815 are two *C. trachomatis* proteins that belong to the eggNOG cluster of orthologous groups COG1109 [**Figure 2A** (Huerta-Cepas et al., 2016)]. Members of this large group of bacterial, archaeal, and eukaryotic proteins display PGM activity and related functions such as phosphomannomutase or phosphoglucosamine mutase activities (Stiers et al., 2017). CT295 (gene annotated as *mrsA_1*) and CT815 (*mrsA_2*) homologs are found among all known chlamydiae. These proteins represent separate monophyletic clades in COG1109 and are related to proteins from diverse bacterial taxa (**Figure 2B**). CT295 and CT815 are only distantly related with each other and thus do not originate from a recent gene duplication event (**Figure 2C**).

**Figure 3:**
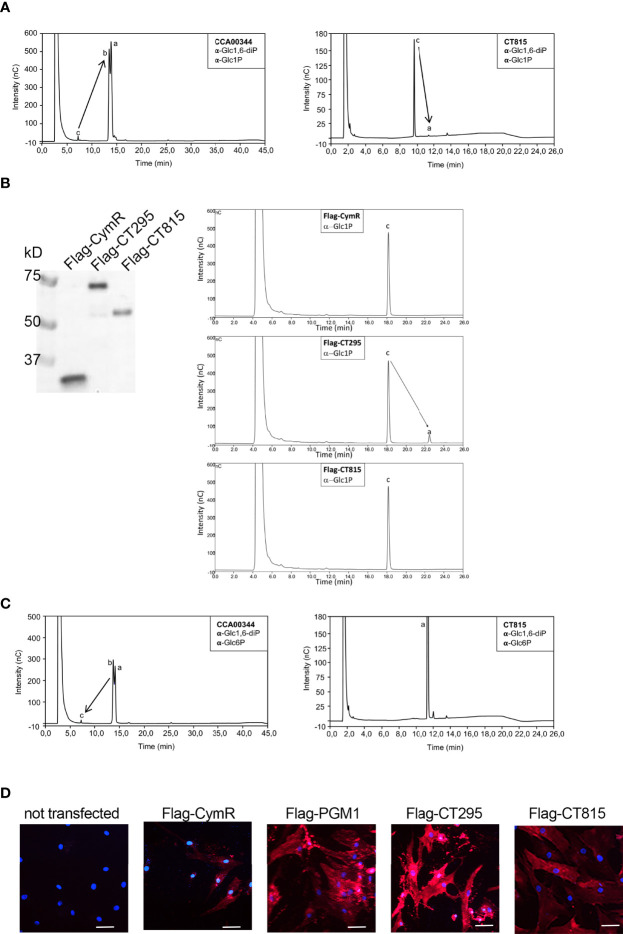
CT295 is a phosphoglucomutase. **(A)** – The indicated purified proteins (bold letters) were incubated with Glc1P in the presence of the co-factor Glc1,6diP. The substrates and reaction products were identified by HPAEC. Incubation of Glc1P (peak c) with CCA00344 resulted in full conversion into Glc6P (peaks a and b), while Glc6P was hardly detectable when CT815 was tested. Note that the samples were run on two different columns, resulting in different elution profiles. **(B)** – The indicated Flag-tagged proteins expressed by transfection in HEK293T cells were immobilized on beads and used to perform enzymatic assay as in A (in the absence of Glc1,6diP) (*right*). A fraction of the transfected cell lysates was analyzed by western blot using anti-Flag antibodies (*left*). **(C)** – The reverse reaction was tested using purified proteins as in A. A very low conversion of Glc6P into Glc1P was observed for CCA00344 and was totally absent for CT815. Of note, CCA00344 favors the conversion of α-Glc6P (peak a) into β-Glc6P (peak b) while CT815 does not. **(D)** – PGM1 deficient fibroblasts were transfected or not with the indicated constructs. One day later, ICAM1 expression at the cell surface was probed with Cy5-coupled anti-ICAM1 antibody (red). Nuclei were stained with Hoechst (blue). Bar is 50 μm.

To test if CT295 and/or CT815 displayed PGM activity we expressed them in *Escherichia coli* with a carboxy-terminal histidine tag. However, CT295-HIS was not expressed by *E. coli*, and after testing the orthologous protein in different *Chlamydiaceae* we chose the *C. caviae* ortholog, CCA00344, which displayed 59% amino acid identity with CT295. CCA00344-HIS and CT815-HIS were purified from *E. coli* extracts (**Figure 3** and **Figure S1A**). As a positive control we also produced the *E. coli* phosphomannomutase CpsG, because our initial attempts to produce the *E. coli* PGM failed, and CpsG turned out to display good PGM activity *in vitro*. To test the ability of these recombinant proteins to convert Glc1P into Glc6P we first incubated them with Glc1P in the presence of glucose 1,6-diPhosphate (Glc1,6diP), a cofactor for the PGM reaction in *E. coli* (Ray and Roscelli, 1964). The samples were incubated for 20 min at 37°C, before stopping the reaction by boiling for 5 min. Sugars present in the samples were analyzed by high performance anion exchange chromatography with pulsed amperometric detection (HPAEC-PAD) (**Figure 3** and **Figure S1B**). Incubation of Glc1P in the presence of CCA00344-HIS led to its full conversion into Glc6P, while only low levels of Glc6P were detected in the presence of CT815-HIS (**Figure 3A**). Interestingly we noted that while we confirmed that CpsG-HIS required the presence of Glc1,6diP to convert Glc1P into Glc6P, CCA00344-HIS did not, indicating that the chlamydial enzyme evolved to bypass co-factor requirement (**Figure 3** and **Figure S1C**). Also, Glc6P appeared as two peaks (a and b in **Figure 3A**), which correspond to the stereoisomers α-Glc6P and β-Glc6P. This indicated that CCA00344-HIS favored the conversion of α-Glc6P into β-Glc6P, a property that we did not observe in CT815 nor CpsG (**Figure 3** and **Figure S1C, D**).

To circumvent the lack of expression of CT295 in the bacterial system, we turned to a mammalian expression system. We cloned CT295 and CT815 with an amino-terminal Flag tag in a mammalian expression plasmid and expressed it in HEK293T cells. Flag-CymR expression plasmid served as a negative control. One day after transfection the cells were lysed, Flag-tagged proteins were immobilized using beads covalently conjugated to anti-Flag antibodies, and tested for their ability to convert Glc1P into Glc6P. The reaction products were analyzed as described above. Glc6P was only detected in the samples incubated with Flag-CT295 loaded beads, not with Flag-CT815 nor with the negative control (**Figure 3B**). These data demonstrated the ability of CT295 to convert Glc1P into Glc6P.

When we monitored the reverse reaction, supplying Glc6P and following the appearance of Glc1P, very low amounts of Glc1P were detected after incubation with CCA00344-HIS or CpsG-HIS, and none with CT815-HIS (**Figure 3C** and **Figure S1D**). This indicated that, *in vitro*, the conversion of Glc1P into Glc6P was more favorable than the reverse reaction. To confirm that CT295 was nonetheless capable of catalyzing the reverse reaction, we made use of human fibroblasts deficient for PGM1. One of the phenotypes associated with PGM1 deficiency is a defect in protein glycosylation, due to the inability to convert Glc6P into Glc1P to feed UDP-Glc synthesis and thereby protein glycosylation (Tegtmeyer et al., 2014). As a consequence, several glycosylated proteins like the cell-surface glycoprotein intercellular adhesion molecule 1 (ICAM-1) are hardly expressed at the cell surface in PGM1 deficient cells (Tegtmeyer et al., 2014). ICAM-1 expression can thus be used as a read-out for PGM activity. As expected, ICAM-1 was hardly detected at the surface of PGM1 deficient fibroblasts (**Figure 3D**). Expression of Flag-CymR had a marginal effect on ICAM-1 expression. In contrast, as expected, transfection of the wild-type human PGM1 restored ICAM1 expression in the PGM1 deficient fibroblasts. Transfection of CT295 also restored ICAM-1 expression, confirming its ability to catalyze the conversion of Glc6P into Glc1P. Transfection of CT815 also resulted in increased ICAM-1 expression, but to a lesser extent, supporting the hypothesis that while CT815 displays some PGM activity, it is likely that it uses a different sugar than glucose as a favorite substrate (**Figure 3D**).

Altogether, these data demonstrate that CT295, and its ortholog in *C. caviae*, are *bona fide* PGMs.

### CT295 Is a T3S Substrate

**Figure 4:**
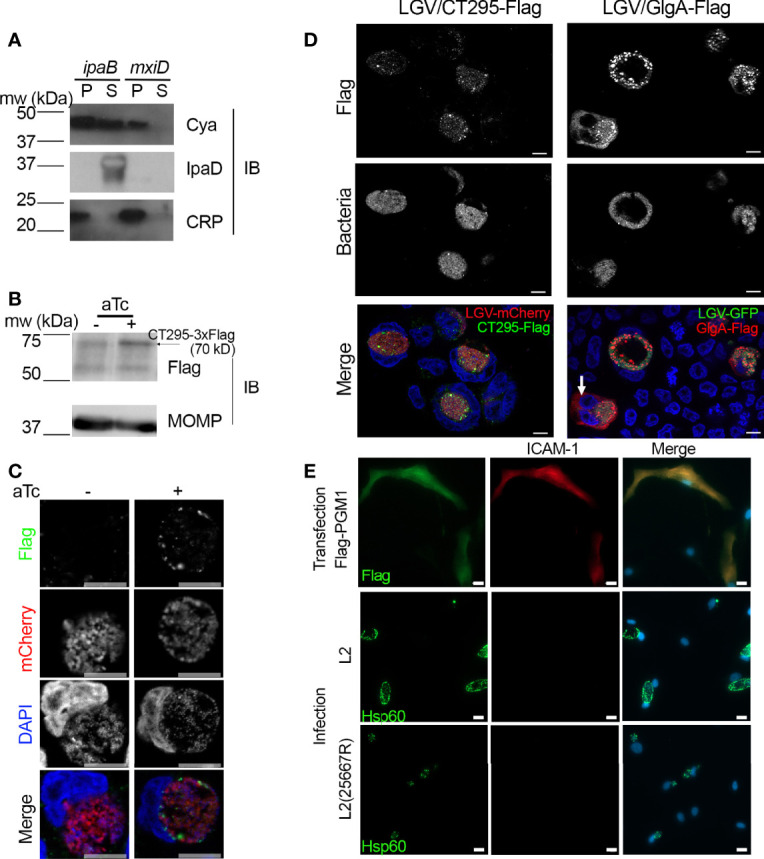
CT295 amino-terminal sequence is recognized by the T3S machinery of *S. flexneri*. **(A)** – The first 22 amino acids of CT295 were fused to the Cya reporter protein and expressed in a S*. flexneri ipaB* (constitutive T3S) or *mxiD* (defective T3S) strain. Exponential phase cultures expressing the reporter fusion protein were fractionated into supernatants (S) and pellets (P). Samples were resolved by SDS-PAGE, transferred to a PVDF membrane, and probed with anti-Cya (to detect the fusion protein), anti-IpaD (*Shigella* secreted protein), or anti-CRP (*Shigella* non-secreted protein) antibodies. **(B)** – Cells were infected with *C. trachomatis* L2 strain stably expressing mCherry with CT295-Flag (Tet-inducible promoter) for 40 h in the presence or not of 20 ng/ml anhydrotetracycline (aTc), before lysis and analysis by western blot. Upon induction, a weak band was detected upon aTc induction (arrow), the other bands correspond to background signal. The membrane was reprobed with antibodies against *Chlamydia*’s major outer membrane protein (MOMP) to control for equal loading. **(C, D)** – Cells infected with *C. trachomatis* expressing CT295-Flag in the absence of presence of 20 nM aTc were fixed 24 hpi **(C)** or 40 hpi (**D**, with aTc), permeabilized and stained with anti-Flag antibody. In panel **(D)**, cells infected with *C. trachomatis* L2 strain stably expressing GFP together with GlgA-Flag (endogenous promoter) were used for comparison. The white arrow points to GlgA-Flag detected in the cytoplasm of one infected cell. Bar is 10 μm. **(E)** – PGM1 deficient fibroblasts were transfected with Flag-PGM1 or infected with the indicated *C. trachomatis* L2 strain. Forty-eight hours later the cells were fixed, permeabilized and stained with the indicated antibodies. Nuclei were stained with Hoechst (blue). Bar is 20 μm.

If CT295 is the PGM that converts Glc1P into Glc6P in the inclusion lumen it needs to be secreted out of the bacteria. We have shown that *C. trachomatis* glycogen metabolism enzymes possessed secretion signals recognized by the T3S machinery of *S. flexneri* (Gehre et al., 2016). To explore if this was also true for CT295, we tested for the presence of a functional T3S signal within its amino-terminus using the heterologous secretion assay in *S. flexneri* as described previously (Subtil et al., 2001). Briefly, we fused these amino-terminal sequences to the reporter protein calmodulin-dependent adenylate cyclase (Cya). The chimeric proteins were then expressed in *S. flexneri ipaB* (constitutive T3S) or *mxiD* (deficient in T3S) null strains. When we analyzed the culture supernatant versus the bacterial pellet, we found the Cya reporter in the supernatant of *ipaB* cultures (**Figure 4A**). The same expression pattern was observed for an endogenous *Shigella* T3S substrate, IpaD. Conversely, we found the cAMP receptor protein (CRP), a non-secreted protein, exclusively in the bacterial pellet. This control shows that Cya detection in the supernatant was not due to bacterial lysis. Finally, the Cya reporter was not recovered in the supernatants of the *mxiD* strain, demonstrating that its secretion was dependent on the T3S system.

### Absence of Evidence for CT295 Secretion in the Cell Cytoplasm

We transformed *C. trachomatis* with an expression plasmid constitutively expressing the mCherry protein, as well as CT295 with a carboxy-terminal 3xFlag tag under the control of an inducible promoter, as an initial attempt using the endogenous promoter showed no protein expression. Upon induction with anhydrotetracycline (aTc) a weak band was detected by western blot on whole cell lysates with anti-Flag antibodies at the expected size (**Figure 4B**). By immunofluorescence, anti-Flag antibodies displayed a weak signal with punctate distribution. CT295-Flag was detected in areas also positive for mCherry or DAPI, indicating that only intra-bacterial CT295-Flag was detected. Thus, this approach could not demonstrate the secretion of CT295-Flag in the inclusion lumen, possibly because it is not sensitive enough to detect the enzyme diluted in this large volume.

Since *C. trachomatis* GlgA is detected both in the inclusion lumen and in the cytoplasm (Lu et al., 2013) we considered the possibility that CT295 could be translocated in the host cytoplasm. We did not detect CT295-Flag in the cell cytoplasm at 24 hpi (**Figure 4C**) nor at 40 hpi (**Figure 4D**). Bacteria expressing GlgA-Flag under the control of its endogenous promoter, and constitutively expressing the green fluorescent protein (GFP) were used for comparison. As expected, a cytoplasmic staining was observed for GlgA-Flag, although only in ~ 10% (n=150 infected cells from 2 independent experiments) of the infected cells, possibly due to low sensitivity of the assay (**Figure 4D**).

As an alternative approach to test for CT295 presence in the host cytoplasm we tested whether infection could restore PGM activity in the cytoplasm of PGM1 deficient fibroblasts. Indeed, if CT295 reached the cytoplasm, it might lead to at least a partial recovery of ICAM-1 glycosylation. Cells transfected with Flag-PGM1 served as a positive control. We observed that ICAM-1 expression, which was undetectable at the surface of PGM1 deficient fibroblast, remained absent from the surface of cells infected for 40 h with *C. trachomatis* (**Figure 4E**). One pitfall of this experiment is that *C. trachomatis* L2 takes up UDP-glucose from the host cytoplasm to accumulate glycogen in the inclusion lumen (Gehre et al., 2016). Therefore, the presence of a bacterial PGM in the cytoplasm might not be sufficient to restore ICAM-1 glycosylation. We thus also used the plasmid-less L2(25667R), which is less likely to deplete the host UDP-glucose stores, as it does not accumulate glycogen in the inclusion (Carlson et al., 2008). Infection with L2(25667R) did not restore ICAM-1expression at the cell surface either (**Figure 4E**). These experiments suggest that CT295 is translocated directly into the inclusion lumen, without an intermediate cytoplasmic step.

### Presence of a T3S in CT295 Orthologs Among *Chlamydiaceae* Correlates With Their Capacity to Accumulate Glycogen in the Inclusion Lumen

**Figure 5:**
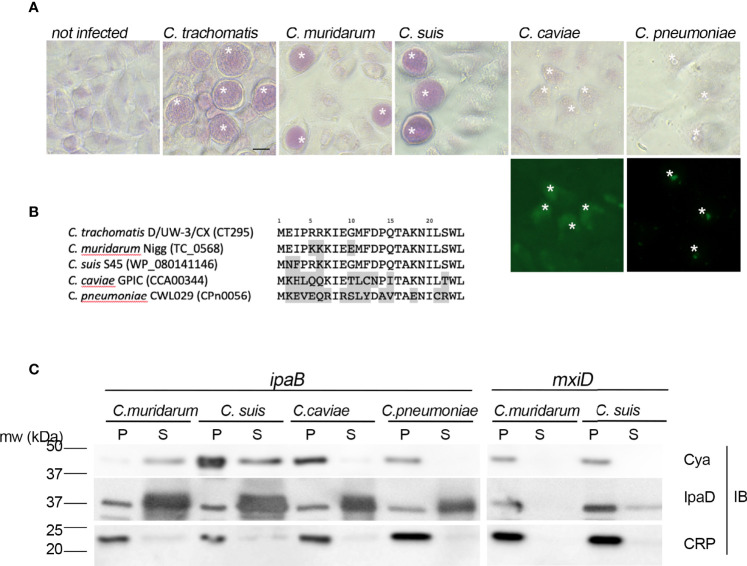
The presence of a functional T3S signal in the PGMs of *Chlamydiaceae* correlates with glycogen storage in inclusions. **(A)** – Top panels: PAS staining of cells infected with the indicated strains. Bottom panels: *C. caviae* and *C. pneumoniae* infected cells were further stained with anti-mGroEL antibody followed with Alexa488-conjugated secondary antibodies to detect the inclusions (asterisks). Bar is 20 μm. **(B)** – Alignment of the first 24 amino acids of CT295 and its orthologs in the indicated *Chlamydiaceae* species. Amino acids that differ from the *C. trachomatis* sequence are shadowed. **(C)** – The first 22 amino acids of the indicated CT295 orthologs were fused to the Cya reporter protein and expressed in a S*. flexneri ipaB* (left) or *mxiD* (right) strains. Secretion was analyzed like in **Figure 4A**.

We next asked whether the secretion of a PGM was a conserved feature among *Chlamydiaceae*. Previous studies have reported glycogen accumulation in *C. suis* and *C. muridarum* inclusions, but the polysaccharide was not observed in the inclusions of more phylogenetically distant *Chlamydia* species such as *C. caviae* or *C. pneumoniae*. We verified this feature by performing periodic acid-Schiff staining (PAS) on cells infected with these different species. Inclusion staining was observed only on cells infected with *C. trachomatis*, *C. suis* and *C. muridarum* (**Figure 5A**). Two amino-terminal amino-acids (W23L24) are conserved in the orthologs of CT295 in all chlamydiae, and the preceding amino acids show low levels of conservation (**Figure 5B**). To test whether these different peptides also function as T3S signals in other species than *C. trachomatis* we constructed chimeras between the amino-terminal 22 amino-acids of the different CT295 orthologs and the Cya reporter gene and tested their secretion when expressed in the *S. flexneri ipaB* strain. The chimera made from the amino-terminal segment of *C. suis* and *C. muriarum* PGMs were recovered in the supernatant, but not those originating from *C. caviae* or *C. pneumoniae*. Secretion was dependent on the T3S system since the chimera were not recovered in the supernatant when expressed by the *S. flexneri mxiD* strain (**Figure 5C**). Thus, the accumulation of glycogen in the inclusion of *Chlamydiaceae* correlates with their ability to secrete a phosphoglucomutase.

## Discussion

To be of metabolic use for the bacteria the glycogen stored in the inclusion lumen needs to be converted into glucose moieties. Glycogen degradation in the inclusion generates Glc1P, and the single glucose transporter of *C. trachomatis*, UhpC, displays transport activity exclusively towards Glc6P. We provide here strong evidence that this conundrum is solved by the secretion of a bacterial PGM, CT295, by the T3S system. Host PGM1 was not translocated into the inclusion lumen, at least not at levels detectable by immunofluorescence. Silencing *pgm1* did not affect the generation of infectious bacteria. Thus, while we cannot exclude that imported host PGM1 also contributes to Glc1P to Glc6P conversion in the inclusion lumen, our data suggest that such activity would only be marginal compared to that of secreted CT295.

*In vitro* and *in cellulo* assays demonstrated that CT295 and its ortholog in *C. caviae* displayed *bona fide* PGM activity. Interestingly, unlike what had been observed in *E. coli* (Ray and Roscelli, 1964), the chlamydial activity did not require the co-factor glucose 1,6-diPhosphate (Glc1,6diP). This may represent an adaptation to the inclusion environment, where this metabolite might be absent. The second *C. trachomatis* enzyme with predicted PGM activity, CT815, diverged from CT295 early in the evolution of the phylum, possibly even before its emergence. It displayed a poor ability to interconvert Glc1P and Glc6P *in vitro* and *in vivo*, indicating that its preferred substrate is different, possibly phosphoglucosamine, as shown for at least one member or the same clade (Jolly et al., 1999).

Transcriptomic studies detected transcription of *ct295* 8 hpi, a timing that coincides with the start of expression of the Glc6P transporter *uhpC* (Belland et al., 2003). This observation is at odds with proteomics data showing that CT295 is abundant in the infectious form, the EBs that accumulate at the end of the developmental cycle, but is hardly detected in the replicative form of the bacteria, the RBs (Saka et al., 2011). Put together, these observations support the hypothesis that most CT295 synthesized by RBs is secreted, and thus absent from purified RBs. The absence glycogen in RBs (Gehre et al., 2016) is also consistent with CT295 being secreted out of the bacteria at that stage of the developmental cycle. Like other T3S substrates, secretion of CT295 probably stops during conversion to the EB stage, and the PGM activity is then mostly exerted inside the bacteria, where it is likely used for the conversion of Glc6P into Glc1P, which serves as a substrate for the ADP-Glc pyrophosphorylase GlgC to initiate glycogen synthesis in the bacteria. One limitation of our work is that LGV L2 strains stably transformed to express Flag-tagged CT295, under the control of an inducible promoter, produced a protein of low abundance, that we could only detect in the bacteria, and not in the lumen of the inclusion nor in the host cytoplasm. So far, bacterial proteins that were convincingly detected by immunofluorescence in the inclusion lumen were proteins forming globular structures (da Cunha et al., 2017; Lei et al., 2021). Glc1P, CT295’s substrate, is presumably free in the inclusion lumen. Therefore, CT295 is not expected to cluster in the inclusion lumen, making its detection by immunofluorescence extremely challenging. However, the presence of a functional T3S signal in *C. trachomatis*, *C. suis* and *C. muridarum*, in spite of differences in their amino-terminal sequence, together with the paucity of the enzyme in RBs, make strong arguments for CT295’s secretion in the inclusion lumen by a type 3 mechanism.

Finally, we made the intriguing observation that chlamydial PGMs possess secretion signals recognized by the T3S machinery of *S. flexneri* only in species that accumulate glycogen in the inclusion lumen. These species are phylogenetically apart from other *Chlamydiaceae*, and differ by several other features (Bush and Everett, 2001). We lack sufficient information on the translocation, in the inclusion lumen or in the host cytoplasm, of glycogen metabolism enzymes of *Chlamydiaceae* other than *C. trachomatis* to draw solid hypotheses on the evolutionary path that led to this situation, we can however try to elaborate on this observation. We have shown that T3S signals were present in glycogen metabolism enzymes of several environmental chlamydiae, indicating that the ability to take control of glycogen storage capacity of the host was acquired early upon evolution of the phylum (Ball et al., 2013; Collingro et al., 2020). One possibility is that the acquisition of a T3S signal in the PGM of the *Chlamydia* lineage (as opposed to species of the formerly called *Chlamydophila* lineage, e.g., *C. pneumoniae* and *C. Caviae*) opened the way to a positive selection of the capacity to store glycogen in the inclusion. Indeed, once Glc1P became convertible to Glc6P, it might have become advantageous to store a lot of glycogen, possibly to compete out the rest of the microbiota, or to weaken host cell defense, as previously discussed (Gehre et al., 2016). Of note, expression of the glycogen synthase GlgA is the limiting factor for glycogen accumulation in the inclusion (Gehre et al., 2016). In *C. trachomatis*, this gene is under transcriptional control by the *C. trachomatis* plasmid (Carlson et al., 2008). Acquisition of this transcriptional control was likely also an important step along the path towards the glycogen storage strategy.

One question left open is whether a more direct mechanism for Glc6P supply in the inclusion lumen of *C. trachomatis* also takes place? An alternative mechanism must exist at least in the *Chlamydiaceae* of the former *Chlamydophila* lineage, considering the absence of glycogen and phosphoglucomutase in their inclusions, and the fact that UhpC transports only Glc6P also in *C. pneumoniae* (Schwoppe et al., 2002). The situation is different in environmental chlamydiae because, unlike *Chlamydiaceae*, environmental chlamydiae express a hexokinase activity (Horn et al., 2004; Omsland et al., 2014; Dharamshi et al., 2020), and are likely able to import glucose, as demonstrated in the case of *Protochlamydia amoebophila* (Sixt et al., 2013).

In conclusion, CT295 as well as GlgA, GlgB and other glycogen metabolism enzymes are all “housekeeping” chlamydial proteins that acquired T3S signals (this study and (Gehre et al., 2016)). Evolution of housekeeping enzymes into type 3 secretion substrates has seldom been investigated in other bacteria, but was also observed for glyceraldehyde-3-phosphate dehydrogenase in enteropathogenic *E. coli* (Aguilera et al., 2012). In that case, the broad range type 3 secretion chaperone CesT was involved, and whether such a chaperone is required for the secretion of chlamydial enzymes remains to be investigated. In any case, the secretion of bacterial enzymes opens the door to a direct modification of the metabolism of the host cell to the benefit of the bacteria, and might therefore occur more frequently than currently known.

## Material and Methods

### Cells and Bacteria

HeLa and HEK293T cells (ATCC) were cultured in Dulbecco’s modified Eagle’s medium (DMEM) with Glutamax (Gibco™ #31966), supplemented with 10% (v/v) fetal bovine serum (FBS). Fibroblast CDG_0072 deficient in PGM1, a kind gift from Pr. Hudson Freeze (La Jolla, CA), were grown in DMEM (Gibco™ # A1443001) supplemented with 1 g/L glucose, 584 mg/mL L-glutamine, 110 mg/mL sodium pyruvate, and 10% FBS. Cells were routinely checked for absence of mycoplasma contamination by PCR. *C. trachomatis* LGV serovar L2 strain 434/Bu (ATCC), the plasmid-less strain LGV L2 25667R (Peterson et al., 1990) and GFP-expressing L2 (L2^IncD^GFP) (Vromman et al., 2014) were purified on density gradients as previously described (Scidmore, 2005). Other *Chlamydia* strains used were *C. suis* SWA107, a kind gift from Nicole Borel (Zürich, Switzerand), *C. pneumoniae* CWL029, *C. muridarum* MoPn and *C. caviae* GPIC. The *Shigella flexneri ipaB* and *mxiD* strains are derivates of M90T, the virulent wild-type strain, in which the respective genes (*ipaB* and *mxiD*) have been inactivated (Allaoui et al., 1993). The *Escherichia coli* strain *DH5α* was used for cloning purposes and plasmid amplification. Both *S. flexneri* and *E. coli* strains were grown in Luria-Bertani (LB) medium.

### Phylogenetic Analysis of CT295 and CT815

Protein sequences of CT295 and CT815 were analyzed with eggNOG v4.5.1 (Huerta-Cepas et al., 2016). They are both members of COG1109 at the last universal common ancestor (LUCA) taxonomic level (including all domains of life). At the bacteria-specific eggNOG clustering, CT295 and CT815 belong to two distinct EggNOGs, ENOG4107QSU and ENOG4107QJF. To identify closely related sequences, we used eggNOG-mapper v1.0.1 (Huerta-Cepas et al., 2017) with the bacteria optimized database using the “–database bact” option and default settings on a broad selection of published chlamydial genomes. We downloaded all COG1109 sequences (n=4,829; http://eggnogapi45.embl.de/nog_data/text/fasta/COG1109), added all chlamydial ENOG4107QSU (n=104) and ENOG4107QJF (n=69) sequences and aligned them using the COG1109 hidden markov model (http://eggnogapi45.embl.de/nog_data/file/hmm/COG1109). To reduce redundancy we clustered the alignment with the cd-hit program (Fu et al., 2012) at 80% sequence identity over at least 90% of the shorter sequence (“-aS 0.9 -c 0.8”). We performed *de novo* multiple sequence alignment of the clustered sequences (n=2,869) with MAFFT 7.222 (Katoh et al., 2002) using the “–localpair” and “–maxiterate 1000” parameter and trimmed the alignment with trimAl “-gappyout” (Capella-Gutiérrez et al., 2009). Only sequences with a gap-rate < 50% were retained (n=2,829). We performed model testing under the empirical LG model (Le and Gascuel, 2008), and with the empirical mixture models C10 to C60 (best model: C60+LG+G+F) and maximum likelihood tree reconstruction with IQ-TREE 1.6.2 (Minh et al., 2013). We inferred support values from 1,000 ultrafast bootstrap replicates (Hoang et al., 2018) with the “-bnni” option for bootstrap tree optimization and from 1,000 replicates of the SH-like approximate likelihood ratio test (Guindon et al., 2010).We next extracted the sister clade of CT295 (n=93) and used it as an outgroup for a more detailed analysis of CT295 related proteins. Sequence alignment, trimming, and initial phylogenetic reconstruction (best model: C60+LG+G+F) was performed as described above. We then used the resulting best tree as a guide tree for the posterior mean site frequency (PMSF) model (Wang et al., 2018) for improved site heterogeneity modeling under C60+LG+G+F and inferred 100 non-parametric bootstraps. Phylogenetic trees were visualized and edited using the Interactive Tree Of Life v4 (Letunic and Bork, 2019).

### Cloning

Primer sequences and cloning methods are described in **Figure S2**, all constructs were verified by sequencing. The gene *ct815* was cloned as follows: the gene was amplified with primers 1 and 4, and with primers 2 and 3. PCR products were mixed, heated to 65°C for 10 minutes, and annealed at 37°C for 30 minutes. The mix was then cloned into the pET28a previously digested by NcoI and XhoI restriction enzymes. Chimeras to test for the presence of T3S signal were constructed like in (Subtil et al., 2001), using the first 22 codons of the gene of interest. Flag-GlgA is described in (Gehre et al., 2016) and the negative control, CymR, that corresponds to a bacterial repressor for gene expression from *Pseudomonas putida* (Mullick et al., 2006) was constructed through Gateway cloning like Flag-PGM1 and PGM1-Flag. For GlgA, cloning was performed in pBOMB4 (Bauler and Hackstadt, 2014) using the endogenous promoter of the *ctl0167* gene, and the construct was transformed into the LGV L2 25667R strain. The CT295-Flag construct was built by overlap PCR and inserted by Gibson reaction into p2TK2(spect)-SW2-mCh(Gro)-NotI-3xFlag-IncDTerm, a kind gift of Isabelle Derré. Briefly, a fragment containing the Tet repressor, the Tet promotor, and the 5’ part of *ct295* was amplified by PCR using p2TK2(spect)-SW2-mCh(Gro)-Tet-IncV-NotI-3xFlag-IncDTerm as template (Stanhope et al., 2017). A second fragment covering the whole *ct295* sequence was amplified using *C. trachomatis* serovar D as template. The two fragments were joined by overlap PCR and inserted by Gibson reaction into p2TK2(spect)-SW2-mCh(Gro)-NotI-3xFlag-IncDTerm digested with NotI and NdeI. The resulting plasmid allows for constitutive expression of mCherry under the control of the groESL promoter and terminator and the inducible expression of the CT295-Flag under the control of the aTc-inducible promoter. It was stably transformed in *C. trachomatis* L2 as described (Wang et al., 2011).

### Production and Purification of Recombinant Proteins

*E*. *coli* BL21 (DE3) was used to produce recombinant CCA00344, CT815, and CpsG harboring a polyhistidine tag at the C-terminal part of the protein. Bacteria were grown at 37°C to an optical density at 600 nm of 0.9 in 2 liters of LB with 30 µg/mL of kanamycin under shaking. 0.5 mM of isopropyl-β-D-thiogalactopyranoside (IPTG) was added and incubation was continued for 18 h at 16°C. Bacteria were harvested by centrifugation, and proteins were purified from clarified lysate by affinity chromatography on Ni^2+^-nitrilotriaceta-agarose (Qiagen) in 20 mM Tris-HCl 500 mM NaCl pH 8.0 buffer and by size exclusion chromatography on Superdex 200 HL 16/600 column (GE Healthcare) in 20 mM Tris-HCl 500 mM NaCl pH 7.0 buffer. All purified proteins were stored at 4°C.

HEK293T cells were used for production of Flag-tagged constructs. Cells were transfected with the indicated plasmids using the JetPrime kit (Polyplus Transfection) according to the manufacturer recommendations. Twenty-four hours post transfection, cells were lysed for 15 min at 4°C in lysis buffer [0.05 M Tris, 0.15 M NaCl, 5% Glycerol and 0.5% Nonidet P-40, pH = 7.5 supplemented with protease inhibitors (Roche)]. Samples were centrifuged at 12 000 x*g* for 10 min, the supernatants were collected and diluted 1:10 in lysis buffer except that Nonidet P-40 was brought to 0.05%. A fraction of the lysates was kept for analysis of the expression of the proteins by western blot. Anti-flag beads (Sigma) washed in the 0.05% Nonidet P-40 lysis buffer were added to the lysates and incubated for 2h at 4°C. The beads were then washed three times in 10 mM Tris-HCl, 0.5mM MgSO4 (pH 8.0) and used immediately for the PGM activity assay.

### Phosphoglucomutase Activity Assay

HIS-tagged CCA00344, CT815 or CpsG (0,1 mg/mL) were incubated for 20 minutes at 37°C with 0.2 mg/mL of glucose-1,6-diphosphate and either 0.66 mg/ml Glc1P or 0.33 mg/ml Glc6P in 10 mM Tris-HCl, 0.5 mM MgSO_4_ buffer (pH 8.0) and then boiled for 5 minutes to stop the reaction. For the reactions using Flag-tagged proteins 0.66 mg/ml Glc1P was added on the beads in 10 mM Tris-HCl, 0.5mM MgSO4 (pH 8.0) in a 0.1 ml reaction volume, and incubated for 20 min at 37°C. The reaction was then stopped by boiling the samples for 5 min. Reaction products were analyzed by high performance anion exchange chromatography with pulsed electrochemical detection (Dionex, model ICS3000). The samples were injected into a CarboPAC-PA1 column pre-equilibrated for 20 minutes with 88.8% of 100 mM NaOH (eluant A) and 11.2% of 100 mM NaOH 720 mM NaOAc (eluant B). After injection, a gradient run (flow rate of 0.350 mL.min^-1^) was performed as follows: 0-2 min 88.8% A + 11.2% B, 2-20 min 88.8% A + 11.2% B – 65% A + 35% B, 20-35 min 65% A + 35% B – 40% A + 60% B, 35-37 min 40% A + 60% B – 100% B. The data displayed are representative of at least 2 independent enzymatic assays. Different columns were used in this study; therefore, retention times were calibrated for each using pure Glc1 and Glc6P.

### Transfections, Immunofluorescence, and PAS Staining

Cells were transfected with the indicated plasmids using the JetPrime kit (Polyplus Transfection) according to the manufacturer recommendations. Twenty-four hours post transfection, cells were fixed with 2% (w/v) paraformaldehyde (PFA) 2% (w/v) sucrose in PBS for 30 minutes at room temperature, then washed with PBS twice. For the panel shown in **Figure 1**, a 4% PFA 4% sucrose solution was used for fixation. Cells were blocked with 10 mg/mL bovine serum albumin (BSA) for 30 minutes before being stained with antibodies anti-ICAM-1 coupled to APC (Invitrogen 17.0549.42) at dilution 1/1000. Cells were then washed twice with PBS, permeabilized with 0.05% saponin in PBS and DNA was stained with Hoechst 33342 (Molecular Probes). For the panel shown in **Figure 4C** the cells were permeabilized after ICAM-1-APC staining to probe for Flag (rabbit, Sigma F7425) or Hsp60 (rabbit, homemade) followed with A488-conjugated secondary antibodies. For the panel shown in **Figures 1**, **4B**, the cells were permeabilized in 0.05% saponin in PBS and stained with mouse anti-Flag M2 antibody (Sigma) and homemade rabbit anti-Cap1 antibody, followed with species-specific secondary antibodies. Images were acquired on an Axio observer Z1 microscope equipped with an ApoTome module (Zeiss, Germany) and a 63× Apochromat lens. Images were taken with an ORCA-flash4.OLT camera (Hamamatsu, Japan) using the software Zen. All images shown are representative of observations made on at least 3 biological replicates, in which more than 50 cells were observed each time.

For periodic-acid-Schiff (PAS) stain cells were fixed in 4% PFA 4% sucrose in PBS for 30 min at room temperature, permeabilized for 5 min in 0.05% saponin in PBS with 1 mg/ml BSA, and staining was performed as described (Schaart et al., 2004). Briefly, cells were incubated in 1% periodic acid (Sigma) for 5 min. Thereafter coverslips were put in tap water for 1 min, quickly rinsed in mQ-H_2_0 and then applied to Schiff reagent (Sigma) for 15 min at room temperature. Afterwards the coverslips were rinsed again in mQ-H_2_0, incubated in tap water for 10 min followed by an incubation step in PBS for 5 min. Periodic acid oxidizes the vicinal diols in sugars such as glycogen to aldehydes, which now in turn react with the Schiff reagent to give a purple colour. Coverslips infected with *C. caviae* and *C. pneumoniae* were further stained with home-made rabbit antibody against *C. muridarum* GroEL, that cross-reacts with the orthologous proteins in these species, followed with Alexa488-conjugated secondary antibodies. Images were acquired using transmission light or green fluorescence excitation on an Axio Vert.A1 microscope (Zeiss, Germany) and an LD A-Plan 40x lense. Images were taken with an AxioCam ICc 1 camera using the software Zen. All images shown are representative of observations made on at least 2 biological replicates, in which more than 50 inclusions were observed each time.

### Progeny Assays

For siRNA transfection 50 000 cells were plated at J0 in a 24-well plate and immediately mixed with Lipofectamine RNAiMAX (Invitrogen) following the manufacturer’s recommendation, using 10 nM of siRNA (see **Figure S2** for sequences). Cells treated with siRNA for 24 h (J1) were infected with L2^IncD^GFP bacteria at a MOI = 0.2. Transfection of siRNAs was repeated on J2. On J3 (48 hpi), cells were detached, lysed using glass beads and the supernatant was used to infect new untreated cells plated the day before (100 000 cells/well in a 24-well plate), in serial dilution. The next day, 3 wells per condition with an infection lower than 30% (checked by microscopy) were detached, fixed and analyzed by flow cytometry to determine the bacterial titer as described (Vromman et al., 2014).

### *Shigella* Heterologous Secretion Assays and Immunoblots

Chimera were constructed as described previously (Subtil et al., 2001) with the primers and templates displayed in **Figure S2**. The sequence of all chimera was verified by sequencing. Analysis of secreted proteins was performed as described previously (Subtil et al., 2001), except that the *ipaB* or *mxiD Shigella flexneri* were co-transformed with the pMM100 plasmid that codes for the *lacI* repressor (selection with tetracyclin) (Jaumouillé et al., 2008) and with the indicated Cya chimera (selection with ampicillin). This was done to avoid the very strong expression of some of the chimeras in the absence of *lacI* repressor. Briefly, 1 ml of a 30°C overnight culture of *S. flexneri ipaB* or *mxiD* transformed with different Cya chimeras was inoculated in 15 ml of LB broth and incubated at 37°C for 1 h. Expression of the Cya chimeras was induced by adding 10 µM IPTG for all chimera except PGMtracho/Cya which was induced at 100 µM IPTG. Bacteria were harvested by centrifugation 3 h later and the supernatant was filtered through a Millipore filter (0.2 μm). To precipitate the proteins 1/10 (v/v) of trichloroacetic acid was added to the supernatants. The pellet fraction and the supernatant fraction (concentrated 40-fold compared to the pellet fraction) were resuspended in sample buffer for SDS-PAGE, transferred to Immobilon-P (PVDF) membranes and immunoblotted with the proper primary antibodies diluted in 1X PBS containing 5% milk and 0.01% Tween-20. Primary antibodies used were mouse anti-cya, rabbit anti-CRP and rabbit anti-IpaD antibodies generously given by Drs N. Guiso, A. Ullmann and C. Parsot, respectively (Institut Pasteur, Paris), mouse anti-Flag M2 (Sigma) and anti-MOMP (Argene). Goat anti-mouse and anti-rabbit IgG-HRP (GE Healthcare) were used at 1:10000 dilution. Blots were developed using the Western Lightning Chemiluminescence Reagent (GE Healthcare). The data displayed are representative of at least three independent experiments.

## Data Availability Statement

The original contributions presented in the study are included in the article/**Supplementary Material**. Further inquiries can be directed to the corresponding author.

## Author Contributions

ST and AS conceived the study and designed the research; ST, MN’G, BN, VA and AS performed the experiments; SK and MH performed phylogeny analyses, ST and AS wrote the manuscript with contributions from SK, MH and VA. All authors contributed to the article and approved the submitted version.

## Conflict of Interest

The authors declare that the research was conducted in the absence of any commercial or financial relationships that could be construed as a potential conflict of interest.

## Publisher’s Note

All claims expressed in this article are solely those of the authors and do not necessarily represent those of their affiliated organizations, or those of the publisher, the editors and the reviewers. Any product that may be evaluated in this article, or claim that may be made by its manufacturer, is not guaranteed or endorsed by the publisher.

## Supplementary Material

Supplementary Figure 1
**(A)** – Migration profile of purified proteins. Purified His-tagged proteins were run on SDS-PAGE and stained with Coomassie blue. Molecular weights are indicated. **(B)** – Elution profiles of a-Glc1P (peak c) and a-Glc6P (peak a). **(C)** – CpsG-HIS (top) or CCA00344-HIS (bottom) were incubated with Glc1P in the presence (left) or absence (right) of the co-factor Glc1,6diP. The substrates and reaction products were identified by HPAEC-PAD. CpsG required the presence of Glc1,6diP for optimal conversion of Glc1P into Glc6P, while CCA00344 did not. Also, CCA00344 favored the conversion of a-Glc6P (peak a) into b-Glc6P (peak b), while CpsG did not. D- CpsG-HIS was incubated with Glc6P in the presence of Glc1,6diP. Conversion of Glc6P into Glc1P was hardly detectable.

Supplementary Figure 2 Oligonucleotides used.
