# Supplementary figures and images for: CT295 Is Chlamydia trachomatis’ Phosphoglucomutase and a Type 3 Secretion Substrate
Source: Front Cell Infect Microbiol. 2022 Jun 20;12:866729. doi: 10.3389/fcimb.2022.866729 (PMC9251005; doi:10.3389/fcimb.2022.866729)

Figure S1

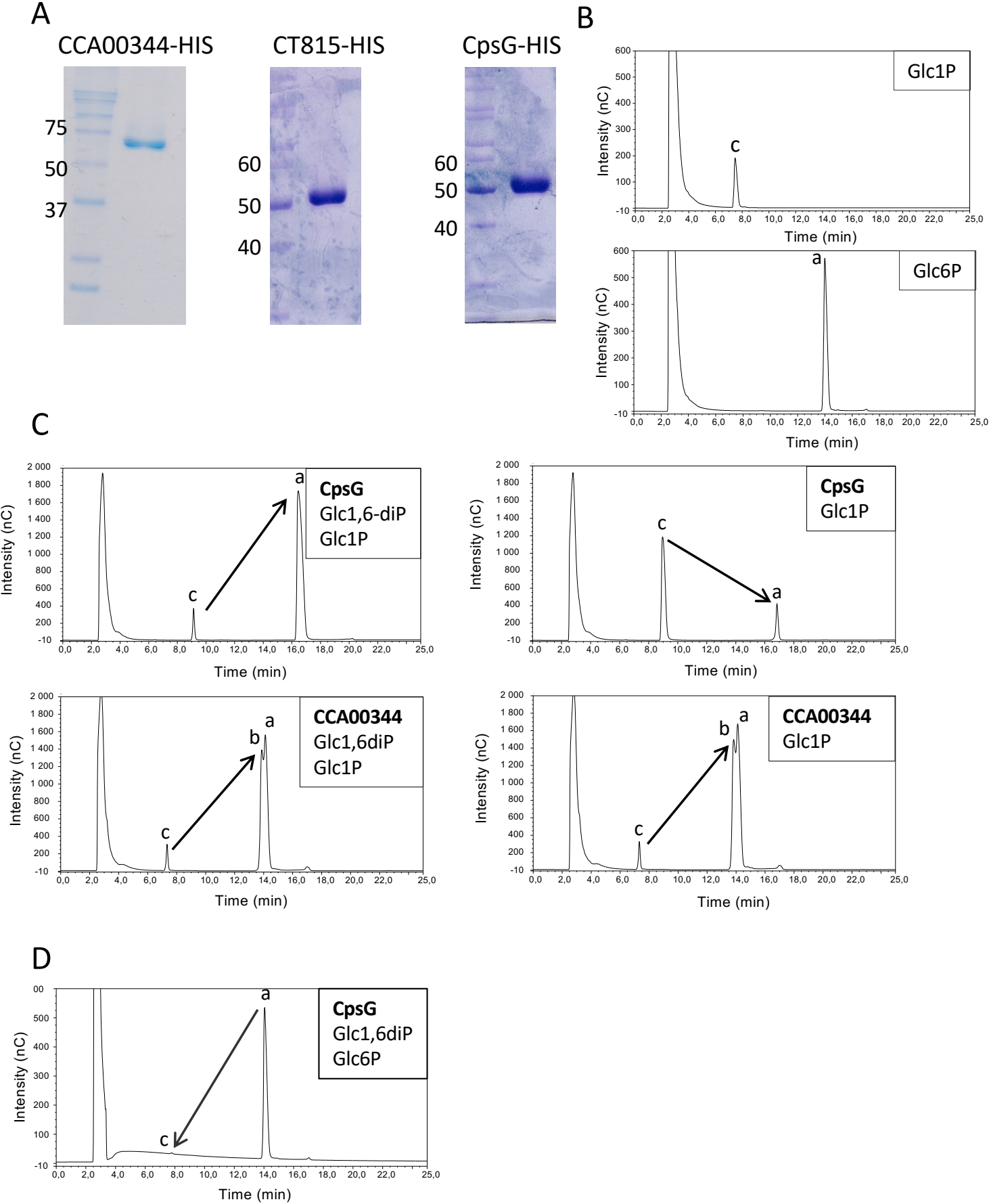

Supplement: Supplementary Figure 1 — (A) – Migration profile of purified proteins. Purified His-tagged proteins were run on SDS-PAGE and stained with Coomassie blue. Molecular weights are indicated. (B) – Elution profiles of a-Glc1P (peak c) and a-Glc6P (peak a). (C) – CpsG-HIS (top) or CCA00344-HIS (bottom) were incubated with Glc1P in the presence (left) or absence (right) of the co-factor Glc1,6diP. The substrates and reaction products were identified by HPAEC-PAD. CpsG required the presence of Glc1,6diP for optimal conversion of Glc1P into Glc6P, while CCA00344 did not. Also, CCA00344 favored the conversion of a-Glc6P (peak a) into b-Glc6P (peak b), while CpsG did not. D- CpsG-HIS was incubated with Glc6P in the presence of Glc1,6diP. Conversion of Glc6P into Glc1P was hardly detectable. [file DataSheet_1.pdf]
