# Supplementary material for: CT295 Is Chlamydia trachomatis’ Phosphoglucomutase and a Type 3 Secretion Substrate
Source: Front Cell Infect Microbiol. 2022 Jun 20;12:866729. doi: 10.3389/fcimb.2022.866729 (PMC9251005; doi:10.3389/fcimb.2022.866729)
Supplement: Supplementary Figure 2 — Oligonucleotides used. [file DataSheet_2.pdf]

Fig. S2: Oligonucleotides and plasmids used

Construct name	Gene	DNA Template	Vector	Tag	Primers	Restriction sites	Cloning method	Resistance
CpsG-HIS	*cpsG*	*E . coli* BW25113	pET28a	6xHis	5'-AAAACCATGGGGAAAAAATTAACCTGCTTTAA-3'<br>5'-TTTTTCTCGAGCTCGTTCAGCAACGTCAGCA-3'	NcoI-XhoI	Restriction enzyme	Kanamycin
CCA00344-HIS	*cca00034*	*C . caviae* GPIC	pET28a	6xHis	5'-TCTAGAAATAATTTTGTTTAACTTTAAGAAGGAGATATACCATGAAGCATCTTCAACAGAA-3'<br>5'-TTTGTTAGCAGCCGGATCTCAGTGGTGGTGGTGGTGGTGCTCGAGTTCAGGCTTTAAAGAAGAGA-3'	NcoI-XhoI	Gibson assembly	Kanamycin
CT815-HIS	*ctl0187*	*C. trachomatis L2/434/Bu*	pET28a	6xHis	5'-CATGGGGACACGTGATGTGAGCCAGCT-3' (1)<br>5'-CGGGACACGTGATGTGAGCCAGCT-3' (2)<br>5'-TCGAGTTCGGAAATCCCGGCTCCAA-3' (3)<br>5'-GTTCGGAAATCCCGGCTCCAA-3' (4)	NcoI-XhoI	See main text	Kanamycin
Flag-CT295	*ctl0547*	*C. trachomatis L2/434/Bu*	pDEST-pCINeoFlag	Flag (Nter)	5'-GGGGACAAGTTTGTACAAAAAAGCAGGCTTGATGGAAATTCCTAGAAGGA-3'<br>5'-GGGGACCACTTTGTACAAGAAAGCTGGGTCTTACTACTAAGAGTAAAAAAGGTGAC-3'	NA	Gateway cloning	Ampicillin
Flag-CT815	*ctl0187*	*C. trachomatis L2/434/Bu*	pDEST-pCINeoFlag	Flag (Nter)	5'-GGGGACAAGTTTGTACAAAAAAGCAGGCTTGATGACACGTGATGTGAGCCA-3'<br>5'-GGGGACCACTTTGTACAAGAAAGCTGGGTCTTACTATTATTCGGAAATCCCGGCTC-3'	NA	Gateway cloning	Ampicillin
Flag-CymR	*CymR*	Entry vector was a gift from Y. Jacob, Institut Pasteur	pDEST-pCINeoFlag	Flag (Nter)		NA	Gateway cloning	Ampicillin
Flag-PGM1	*pgm1*	HeLa cells	pDEST-pCINeoFlag	Flag (Nter)	5'-GGGGACAAGTTTGTACAAAAAAGCAGGCTTGATGGTGAAGATCGTGACAGT-3'<br>5'-GGGGACCACTTTGTACAAGAAAGCTGGGTCTTACTATTAGGTGATGACAGTGGGTG-3'	NA	Gateway cloning	Ampicillin
PGM1-Flag	*pgm1*	HeLa cells	2Flag-pDEST-C Addgene #118372	Flag (Cter)	5'-GGGGACAAGTTTGTACAAAAAAGCAGGCTgccaccATGGTGAAGATCGTGACAGTTA-3'<br>5'-GGGGACCACTTTGTACAAGAAAGCTGGGTcGGTGATGACAGTGGGTGC-3'	NA	Gateway cloning	Ampicillin
PGM_CtrachomatisL2-cya	*ctl0547* (22 codons)	*C. trachomatis L2/434/Bu*	pUC19Cya	Cya	5'-agtcaagcttgTAAagtaccaggagaaattaactATGGAAATTCCTAGAAGGAAAATAGAG-3'<br>5'-agtctctagaAGACAAAATGTTTTTGGCTGTTT-3'	HindIII-XbaI	Restriction enzyme	Ampicillin
PGM_muridarum-cya	*TC_RS02875* (22 codons)	*C . muridarum MoPn*	pUC19Cya	Cya	5'-AGTCaagcttgTAAagtaccAGGAGAaattaactATGGAAATTCCAAAAAAGAAAATTGAGG-3'<br>5'-AGTCtctagaAGATAAAATATTTTTGGCAGTTTGAGG-3'	HindIII-XbaI	Restriction enzyme	Ampicillin
PGM_suis-cya	*WP_080141146* (22 codons)	*C . suis* 4.29b	pUC19Cya	Cya	5'-AGTCaagcttgTAAagtaccAGGAGAaattaactATGAATTTTCCAAGAAAAAAAAATAGAAGGG-3'<br>5'-AGTCtctagaAGAGAGAATATTCCTAGCTGTTTGA-3'	HindIII-XbaI	Restriction enzyme	Ampicillin
PGM_caviae-cya	*cca0034* (22 codons)	*C . caviae GPIC*	pUC19Cya	Cya	5'-AGTCaagcttgTAAagtaccAGGAGAaattaactATGAAGCATCTTCAACAGAAAATAGAA-3'<br>5'-AGTCtctagaGGTTAAGATATTCTTCGCTGTTATAGGG-3'	HindIII-XbaI	Restriction enzyme	Ampicillin
PGM_pneumoniae-cya	*cpn0056* (22 codons)	*C . pneumoniae CWL029*	pUC19Cya	Cya	5'-AGTCaagcttgTAAagtaccAGGAGAaattaactATGAAAGAAGTAGAACAACGTATCCGG-3'<br>5'-AGTCtctagaTCTACAAATATTTTCAGCTGTTACTG-3'	HindIII-XbaI	Restriction enzyme	Ampicillin
CT295-Flag	*ct295*	p2TK2(spect)-SW2-mCh(Gro)-Tet-IncV-NotI-3xFlag-IncDTerm<br>*C . trachomatis* D/UW-3/CX	p2TK2(spect)-SW2-mCh(Gro)-NotI-3xFlag-	3xFlag (Cter)	5'-gcttacgaccatccttttctaccggtggtaccccatggcatatgTTAAGACCCACTTTCACATTTAAG-3'<br>5'-TCTATTTTCCTTCTAGGAATTTCCATttcacttttctctatcactg-3'<br>5'-cagtgatagagaaaagtgaaATGGAAATTCCTAGAAGGAAAATAGA -3'<br>5'-TGATCTTTATAATCACCGTCATGGTCTTTGTAGTCcatGCGGCCGCcagagtaaaaaggtaactcttggtttcttta-3'	NdeI-NotI	overlap PCR followed with Gibson assembly	Spectinomycin
GlgA-Flag	*ctl0167 (glgA)*	*C. trachomatis L2/434/Bu*	pBOMB4	Flag (Cter)	5'-ttatccgcggccgcCCTTCTATGAGCAGAAACTATCCAT-3'<br>5'-cgatgtcgacCTATTACTTGTCGTCATCGTCTTTGTAGTCttgtttataaatttctaaatatttattggcgg-3'	NotI-SalI	Restriction enzyme	Ampicillin

siRNA sequences: *sipgm1*_ 1 CCACAUCCGUGAGAAAGAU dTdT
sipgm1 _2 GCAAGACAAUUGAAGAAUA dTdT
